# Supplementary material for: Effects of an Organic-Inorganic Hybrid Containing Allyl Benzoxazine and POSS on Thermal Properties and Flame Retardancy of Epoxy Resin
Source: Polymers (Basel). 2019 May 1;11(5):770. doi: 10.3390/polym11050770 (PMC6571948; doi:10.3390/polym11050770)
Supplement: Supplementary file 1 [file polymers-11-00770-s001.pdf]

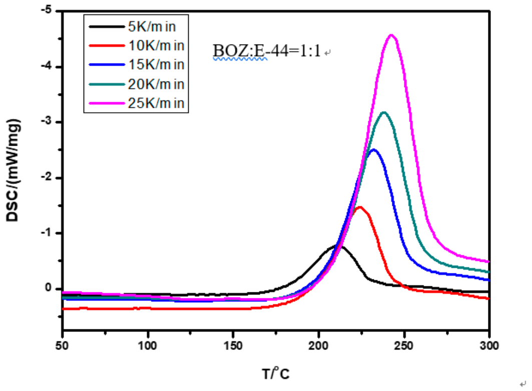

Figure S1. The DSC curves of benzoxazine at different heating rates.

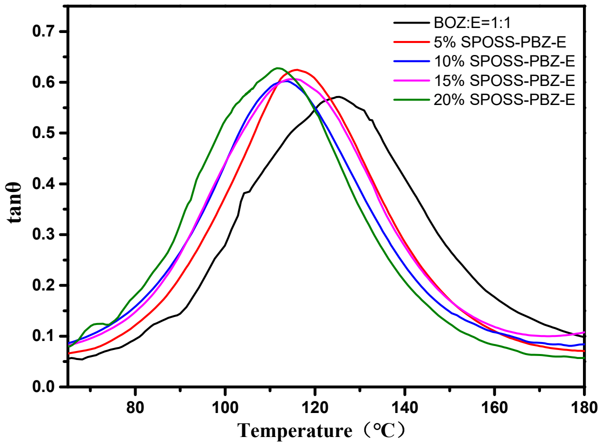

Figure S2. Tan δ curves of nanocomposites containing various SPOSS-BOZ contents.

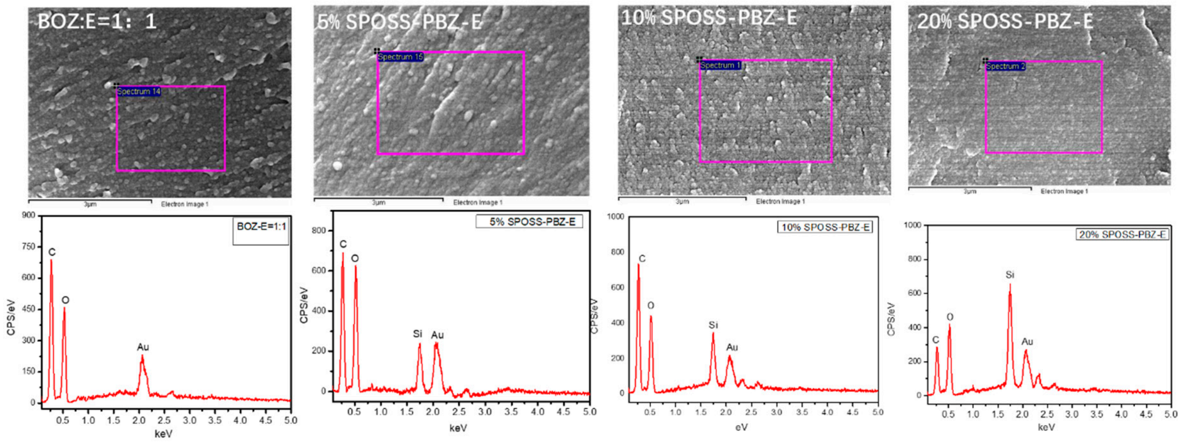

Figure S3. EDX analysis of the fracture surface of SPOSS-PBZ-E.
